# Acceptability of a very‐low‐energy diet in Type 2 diabetes: patient experiences and behaviour regulation

**DOI:** 10.1111/dme.13426

**Published:** 2017-08-15

**Authors:** L. Rehackova, V. Araújo‐Soares, A. J. Adamson, S. Steven, R. Taylor, F. F. Sniehotta

**Affiliations:** ^1^ Department of Health Psychology Newcastle upon Tyne UK; ^2^ Human Nutrition Research Centre Institute of Health and Society Newcastle upon Tyne UK; ^3^ Magnetic Resonance Centre Institute of Cellular Medicine Newcastle University Newcastle upon Tyne UK; ^4^ Fuse the UK Clinical Research Collaboration Centre for Translational Research in Public Health UK

## Abstract

**Aims:**

To evaluate the acceptability of an 8‐week very‐low‐energy diet for remission of Type 2 diabetes, and to identify barriers and facilitators of adherence and behaviour‐regulation strategies used by participants in the Counterbalance study.

**Methods:**

Eighteen of 30 participants in the Counterbalance study (ISRCTN88634530) took part in semi‐structured interviews. Of these, 15 participants were interviewed before and after the 8‐week very‐low‐energy diet intervention. Thematic analysis was used to analyse the narratives.

**Results:**

The prospect of diabetes remission, considerable weight loss, and long‐term health improvement provided participants with substantial initial motivation. This motivation was sustained through the experience of rapid weight loss, improvements in blood glucose levels, social support and increased physical and psychological well‐being. Overall, adherence to the very‐low‐energy diet for 8 weeks was perceived as much easier than anticipated, but required personal effort. Participants addressed challenges by removing food from the environment, planning, avoidance of tempting situations or places, and self‐distraction. Weight loss and improvements in blood glucose levels lead to a sense of achievement and improvements in physical and psychological wellbeing.

**Conclusions:**

Dietary treatment for reversal of Type 2 diabetes is acceptable and feasible in motivated participants, and the process is perceived as highly gratifying. Research outside of controlled trial settings is needed to gauge the generalisability of these findings.

## What's new?

- This is the first qualitative study to explore the acceptability of and patient experiences with very‐low‐energy diets (VLEDs), conducted within a diabetes remission study.
- ‘Before and after’ interviews identified barriers, facilitators and behaviour‐regulation strategies used by the participants and others. These findings can be used to support people on such programmes in the future.
- The findings dispel concerns about the acceptability of VLEDs under clinical supervision, and highlight opportunities to further optimize support during dietary diabetes remission.

**What's new?**

- This is the first qualitative study to explore the acceptability of and patient experiences with very‐low‐energy diets (VLEDs), conducted within a diabetes remission study.
- ‘Before and after’ interviews identified barriers, facilitators and behaviour‐regulation strategies used by the participants and others. These findings can be used to support people on such programmes in the future.
- The findings dispel concerns about the acceptability of VLEDs under clinical supervision, and highlight opportunities to further optimize support during dietary diabetes remission.

## Introduction

The majority of people with Type 2 diabetes are overweight or obese 1, 2. Return to normal blood glucose control can be achieved by substantial weight loss using a very‐low‐energy diet (VLED) 3. A systematic review of the efficacy and acceptability of VLEDs among people with Type 2 diabetes found that VLEDs induce greater weight losses than minimal interventions, standard care or low‐energy diets at 3 and 6 months 4. Although attrition rates, as indicators of acceptability in studies using VLEDs, have improved within the last three decades and are similar to attrition rates in other weight loss interventions 4, 5, 6, 7, 8, 9, few studies have directly assessed the acceptability of VLEDs through improvements in patients’ quality of life 10, 11. Only one qualitative study explored patient experiences with a 3‐month VLED as treatment for obesity, delivered in a group setting 12. That study found that social support and participation in a research study, together with improvements in well‐being achieved during the VLED facilitated weight reduction. The present study aims to further explore experiences, perceived barriers and facilitators of adherence, and behaviour regulation strategies used among people with Type 2 diabetes undertaking a VLED as part of a diabetes remission study (the Counterbalance study) 13.

## Methods

The Counterbalance study (ISRCTN88634530) was a prospective, single‐centre study conducted in the UK. The aim of the study was to achieve normalisation of blood glucose levels (diabetes remission), insulin secretion and insulin action, and reductions in the fat content of the liver and pancreas through weight loss with a VLED. It comprised an 8‐week VLED, followed by a 2‐week stepped return to an isocaloric diet of usual foodstuffs in 30 adult participants diagnosed with Type 2 diabetes for 0.5–23 years. During the 8‐week weight‐loss phase, participants were prescribed consumption of 800 kcal/day in total. Approximately 600 kcal/day came from three sachets of a liquid formula VLED (Optifast) used instead of meals. The VLED consisted of chocolate‐, vanilla‐ and strawberry‐flavoured drinks, a vegetable soup and a dessert in a form of sachets dissolvable in water. The participants were also allowed an additional 240 g of vegetables per day, to equal 800 kcal per day altogether. They were also asked to abstain from alcohol. All oral hypoglycaemic agents were discontinued before the start of the study. The participants were asked to attend the Magnetic Resonance Centre for two main visits during the weight‐loss phase: at baseline, and at week 10. Each of these visits comprised 1.5 days of study. In addition, the participants attended the Magnetic Resonance Centre at weeks 1, 4 and 8 for monitoring of biomedical measures, and to review their experience of the VLED with the Counterbalance study staff and nurses. One‐to‐one support was provided weekly by telephone, e‐mail and face‐to‐face to maximize adherence. Adherence to the VLED was monitored by changes in body mass, food diary and plasma ketone measurements. Participants were asked to measure and record their fasting and post‐meal blood glucose levels three times a week.

The weight‐loss phase was then followed by a structured, individualized weight maintenance programme over 6 months, reported elsewhere 14. The present study is based on semi‐structured interviews conducted before and after completion of the 8‐week VLED (weeks 8–10). During this period, participants were asked to achieve weight loss goals (at least 2.8% body weight loss at week 1 and 8% at week 8) agreed individually in advance. Only those participants who met their individual weight loss goals set by the study staff for weeks 1, 4, and 8 could continue on the study. At the end of the VLED phase, participants’ weight fell from 98.0±2.6 to 83.8±2.4 kg, and 12 out of 30 participants achieved fasting plasma glucose < 7 mmol/l after return to an isocaloric diet, that is, achieved diabetes remission.

### Sampling strategy and participants

The Counterbalance study was advertised using leaflets and by word of mouth and the participants were self‐selected. The inclusion criteria were: diagnosis of Type 2 diabetes of < 4 or > 8 years; HbA_1c_ level < 80 mmol/mol (9.5%); BMI 28–40 kg/m^2^; age 25–80 years; and stable weight in the previous 6 months (within a range of 5 kg). A full list of inclusion and exclusion criteria for the Counterbalance study can be found in the published protocol (ISRCTN88634530). The study commenced in July 2012 and data collection for the present study was initiated in March 2013. Participants were invited to take part by a member of the Counterbalance study team in person (S.S.). Participant details are further presented in Table 1.

**Table 1:** Study participants’ characteristics, interview time points, and observed changes in weight, BMI and blood glucose levels from baseline to follow‐up

Participant number	Gender	Age,years	Type 2 diabetes duration, years	T1	T2	Weight at T1, kg	BMI at T1, kg/m^2^	FPGat T1,mmol/l	Weight loss at T2, kg	% Weight loss at T2, kg	BMI change at T2, kg/m^2^	FPG change at T2, mmol/l	% FPG change at T2, mmol/l
1*	Man	67	3.5	X	X	106	34.6	7.39	–16.90	–15.94	–5.5	–2.25	–30.48
2	Woman	61	11	X	X	87.4	33.1	15.43	–13.00	–14.87	–4.9	–6.79	–44.03
3†	Woman	65	3	X	X	87.4	33.5	11.88	–11.70	–13.39	–4.5	–4.20	–35.33
4*	Woman	42	1		X	112.9	36.4	8.89	–13.30	–11.78	–4.3	–2.64	–29.71
5†	Man	44	2.5	X	X	106.5	36.4	12.65	–12.90	–12.11	–4.4	–5.48	–43.33
6*	Man	54	0.5	X	X	90.9	32.4	6.70	–16.30	–17.93	–5.8	–1.50	–22.35
7†	Man	65	13	X	X	119.80	41.0	13.54	–16.70	–13.94	–5.7	–6.48	–47.87
8	Man	64	9	X		90.00	29.4	11.99	–8.30	–9.22	–2.7	–3.91	–32.59
9*	Man	49	9.5	X	X	97.5	31.8	14.21	–16.70	–17.13	–5.5	–9.16	–64.45
10‡	Man	52	1	X		107.6	34.0	11.5					
11*	Woman	47	2.5	X	X	109.5	39.0	6.93	–17.20	–15.71	–6.1	–1.67	–24.04
12*	Woman	35	1.5		X	102.2	38.0	9.42	–15.00	–14.68	–5.6	–3.97	–42.17
13*	Man	69	8.5	X	X	105.2	37.7	8.84	–18.00	–17.11	–6.5	–5.11	–57.79
14*	Man	59	10	X	X§	96.2	32.9	6.92	–15.20	–15.80	–5.2	–1.98	–28.57
15*	Man	69	3.5	X	X	108.6	33.1	8.45	–22.20	–20.44	–6.8	–3.81	–45.07
16	Woman	64	12	X		119.2	45.7	15.43	–14.90	–12.50	–5.7	1.11	7.19
17*	Woman	70	15	X	X	74.3	31.5	12.54	–8.80	–11.84	–3.7	–5.71	–45.49
18	Man	69	18		X	104.2	32.2	17.32	–14.50	–13.92	–4.5	–5.44	–31.41

VLED, very‐low‐energy diet; FPG, fasting plasma glucose; T1, interview at the beginning of the VLED; T2, interview at the end of the weight loss phase (week 8).

Negative values represent a decrease.

*Participant achieved normal (≤6.9 mmol/l) FPG levels. ^†^Participant achieved near‐normal (ranging from 7.06 to 7.68 mmol/l) FPG levels. ^‡^Participant discontinued the Counterbalance study, and his data were not included in any further analyses.

^§^Interview recording was corrupt due to a technical failure.

### Data collection and saturation

All participants who agreed to take part were interviewed and data saturation was achieved for the reported themes 15.

### Interview protocol

Interview schedules were informed by a multidisciplinary study team, the wider qualitative literature on adherence to weight‐loss treatments and theory‐linked interview approaches 16, 17. They included open‐ended questions and prompts about the participants’ experience with the VLED intervention and were piloted with three independent health psychology researchers (File S1). The interviews were semi‐structured, conducted face‐to‐face and audio‐recorded. Field notes were taken during and after the interviews.

### Consent and interview procedure

Informed consent for the interviews was integrated in the Counterbalance study's consent procedure, approved by Newcastle and North Tyneside 2 Ethics Committee (REC 12/NE/0208). The interviewer (L.R.) was a health psychology doctoral student with a master's degree in health psychology. She had received training in interviewing skills, qualitative methodology and Good Clinical Practice before the beginning of the study and received ongoing supervision during the study by an experienced academic clinical psychologist. The interviewer did not know any of the participants prior to the study commencement. She explained her role in the study, the aims of the interviews and her interest in the study to participants. No incentives were offered for participation.

### Setting

The interviews were conducted in a private screening room on Newcastle University premises or in a clinical room, where a nurse was present. When a nurse was present, permission to conduct the interview was verbally obtained from the participant again.

### Analytical approach

All interviews were anonymized and transcribed verbatim. The analytical strategy aimed to: understand barriers and facilitators to completing an 8‐week VLED within a clinical study; understand how intervention procedures can be optimized to maximise acceptability of this approach; and enable more individuals with Type 2 diabetes to succeed with dietary diabetes remission in the future. We used a thematic approach to data analysis, with coding of predefined theory‐based themes, as well as additional themes identified in the data. The initial themes were drawn from the Theory Domains Framework 18, which had previously been used in a number of exploratory interview studies to identify barriers and facilitators to behaviour 17, 19. An additional domain (VLED evaluation) reflecting evaluations of acceptability of the VLED and the intervention features and suggestions for improvement of the intervention was included in the coding framework. A number of interview sections were independently double‐coded by two coders (L.R., V.A.S.) to ensure consistent coding and data interpretation, and few differences were resolved by discussion. The rest of the data were then coded, sorted by themes and summarized by L.R. Subgroup analyses of transcripts from participants with long vs short duration of diabetes, as well as comparisons of experiences of participants who achieved remission vs those who did not were also conducted. Participants were not invited to comment on the findings. Nvivo v.10 software was used to support the data analysis.

## Results

### Participants

Everyone invited to participate agreed, allowing recruitment of 18 out of 30 participants.

Fifteen participants were interviewed at baseline (T1). One participant was excluded from the Counterbalance study after 2 weeks as a result of not meeting the prespecified weight loss goal. Fifteen participants were interviewed at follow‐up (T2). Three participants were unavailable for the first and three participants were unavailable for the second interview. Table 1 summarizes participant characteristics, attended interviews and physiological outcomes. Reasons for not attending interviews were lack of time, work commitments, and technical issues with scheduling of appointments. One recording of the follow‐up interview was corrupted because of technical failure of the recorder.

The qualitative subgroup analyses did not find substantial differences between remitters and non‐remitters, or differences between participants with short or long duration of diabetes. The results are therefore reported at a group level.

### Interview length

**Figure 1:**
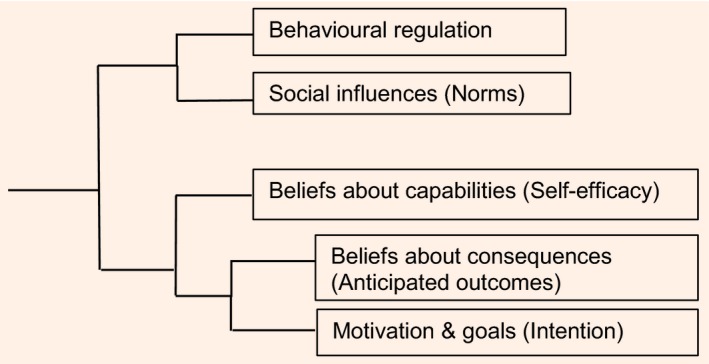
Coding tree, based on an NVivo v.10 cluster analysis of data at baseline (start of the very‐low‐energy diet).

**Figure 2:**
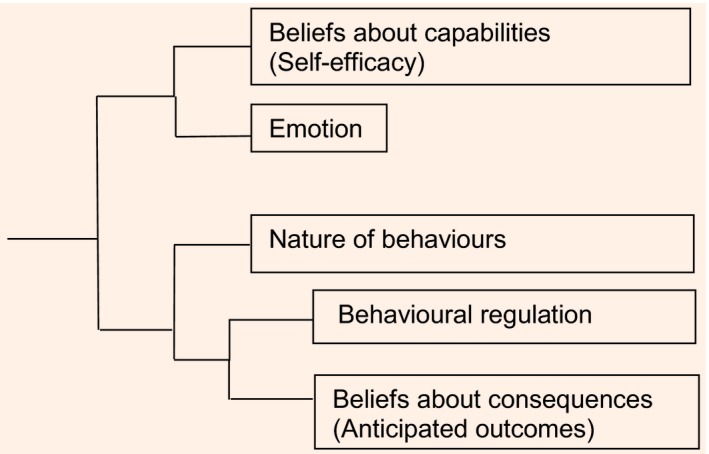
Coding tree based on an NVivo v.10 cluster analysis of data at follow‐up (end of the very‐low‐energy diet).

The median (range) length of the interviews was 24 (12–41) min at T1 and 44 (16–73) min at T2. The main themes coded in the final analyses of interviews at baseline were: motivation and goals; beliefs about consequences; beliefs about capabilities; behaviour regulation; social influences; emotion; and knowledge. Themes coded in transcripts of the follow‐up interviews additionally included: nature of behaviours (e.g. developing a routine); environmental context and resources; and VLED evaluation. Figures 1 and 2 show the coding trees at baseline and follow‐up. Examples of the participants’ narratives related to the themes of VLED evaluation and barriers and facilitators are shown in Tables 2 and 3, respectively.

**Table 2:** Examples of participants’ evaluation of various aspects of the very‐low‐energy diet intervention

Main theme	Sub‐theme	Example quotation
VLED evaluation	Lack of variability of flavours	‘Towards the last week and a half I got bored. Boredom set in but that was all. I mean boredom in the sense of… just lack of variety…’ (Man, aged 69 years, diabetes duration 8.5 years).
	Taste	‘I remember when I had my first milkshake I was like this is going to be horrible. It's going to taste disgusting, it's going to be dead powdery, I'm not going to enjoy it and I remember how pleasantly surprised I was and how creamy it was because the strawberry one it's really creamy.’ (Woman, aged 35 years, diabetes duration 1.5 years)
	Regimen and structure	‘What I found with the diet is that the regimen suits me. I like to know what I'm going to have to eat. If I get choice, if I get here's a shelf full of food go and choose something and potentially I can choose the wrong foods, so if I plan and know what it is that I'm going to eat then I can do it quite easily.’ (Man, aged 49 years, diabetes duration 9.5 years).
	Physical well‐being	‘It was fairly hard to start with but it got easier as the weeks went on and then when I started getting a bit fitter and I could walk further and stand up and sit down and dig the garden it's great now. I feel great.’ (Man, aged 44 years, diabetes duration 2.5 years).
	Psychological well‐being	95. ‘I think as my weight's gone off I think my mood's improved quite a bit. I feel quite, I think because I'm enjoying doing the diet and the research project and I'm looking forward to what's going to happen in the future I think, I don't know, I just feel more lighter.’ (Woman, aged 35 years, diabetes duration 1.5 years).
	Research contact	96. ‘There's the researcher being the person that she is or comes across as anyway, there is no problem if you want to go back to her and that really is enough for me that she's there in the background. If I need to contact her I know that there will be an ear there.’ (Man, aged 67 years, diabetes duration 3.5 years).
	Satisfaction with outcomes	‘…my nurse was practically doing cartwheels, to say the least. My cholesterol has come from 7 point something down to 3.3 so she's taken us [me] off my statins. She said my blood sugar is now that of a normal person, not of a diabetic and in 6 months since I was last there I've lost 3 stone 2 pounds so she was over the moon.’ (Woman, aged 47 years, diabetes duration 2.5 years).
	Suggestions for improvement	I think comparison helps. I mean I've asked questions all the way though about how is it going in the study and it's all of those things isn't it? It's about how am I doing in relation to other people. Am I doing better than other people because that's always nice to know if you are. When I came in after week 1 or week 2 and I'd only lost a very small amount of weight and then that's quite worrying.’ (Man, aged 69 years, diabetes duration 8.5 years)

VLED, very‐low‐energy diet.

**Table 3:** Examples of participants’ narratives related to barriers and facilitators of adherence with the very‐low‐energy diet

Main theme	Sub‐theme	Example quote
Barriers to adherence	Hunger	‘The first week or so I was probably feeling hungry but after that, absolutely fine…I did think Christ, how am I going to manage on three drinks a day, but absolutely fine.’ (Man, aged 69 years, diabetes duration 3.5 years).
	Emotional distress	I had some news on Friday which at the weekend, I got it on Friday but Saturday it hit us like a brick wall and I was like ‘oh, and I really wanted comfort food’ (Woman, aged 47 years, diabetes duration 2.5 years)
	Environment	Seeing cold meat hanging around sometimes, a leg of chicken or a breast and it would be on a plate in the kitchen … At times I would twitch and go oh Sylvia [wife] ‘you've got to move that damn chicken before I go in there’ (Man, aged 67 years, diabetes duration 3.5 years).
Facilitators of adherence	Rapid outcomes	I found it very easy and I got results very quickly. I started to see weight loss fairly quickly and that's encouragement in itself’ (Man, aged 69 years, diabetes duration 18 years).
	Social support	The major support that I had, I knew my family were behind me with regards to it and I could do and ask them anything. My boss has given me a tremendous level of support by giving me the time off work to come here today and stuff like that and just to give me little words of encouragement’ (Man, aged 49 years, diabetes duration 9.5 years)
	Involvement in a study	I thought well there's no point I'm coming here, I'm getting support, I'm getting help and if I don't do – I mean people don't know what you're doing in your own house, but I thought if I'm not honest and stick to it then I won't lose the weight and my blood sugar won't go down so it's just trying to be motivated and to be sensible and think well this is a chance that you've taken, an opportunity to lose weight, opportunity to get your blood sugar down so take it, grab it with both hands’ (Woman, aged 70 years, diabetes duration 15 years).

### Motivation and initial expectations

The prospect of diabetes remission and weight loss were the main motives for taking part in the Counterbalance study, in which the expected individual weight loss was ~15 kg.

Prior to joining the Counterbalance study, many participants had tried to lose and maintain weight with various levels of success. Some of the main reasons for this were slow weight loss and boredom with the weight‐loss regimen or dislike of it, which eventually resulted in weight regain. The appeal of large and quick weight loss was apparent through the participants’ desire to be able to dress in more appealing clothing, become more confident, and feel better about their bodies as a consequence of the weight loss. The participants also anticipated improvements in their long‐term health and regaining control over their health. Ageing and the will to increase the length and quality of life in the future were recognized as important drivers, and were often underlined by the desire to be healthy enough to take care of and spend time with their families later in life.

The main thing that I'm after is trying to cut myself free from diabetes. That's a very strong motivation, because I am not getting any younger…It would be fantastic to come off the end of the study with a clean bill of health, in a situation where I can control my weight at a lower level, knowing that any future illnesses might not be coming my way, because I've taken some action now. (man, aged 49 years, 9.5 years since diagnosis).

Although the participants did not report that diabetes affected their day‐to‐day lives substantially, the practicalities of diabetes management, including taking tablets or experiencing side‐effects associated with them, were the major sources of complaints among people treated with medication.

I hate taking tablets, and I'm on six a day, so… I mean I'll try anything to get rid of it. If it goes, great. If it doesn't, well, at least it's helped. Well, it will have helped. I might come off the tablets, I don't know; just be a diet only.’ (man, aged 44 years, 2.5 years since diagnosis).

It was common for participants to try to identify the potential barriers to adherence before they started the VLED. One of the most anticipated barriers to adherence was negative emotions (e.g. during stressful situations), which could affect participants’ ability to adhere to the VLED and potentially cause a spiral of lapses. Availability of social support was expected to affect adherence too; for example, through approval or disapproval of the participants’ taking part in the study, offering or refraining from offering food, or providing encouragement and information to the participants. The prospect of support from the study staff also affected the participants’ initial motivation to adhere to the diet (because of regular weighing and monitoring) and outcome expectations (through goal‐setting for weight and blood glucose).

### Experience with the VLED

On the whole, adhering to the VLED was perceived as easier than the participants had expected. This was in contrast to their anticipation of hunger and their initially low levels of self‐efficacy in dealing with temptations expressed during the baseline interviews.

I was so surprised, compared to what I was eating to what I have been eating over the last weeks I really would have thought that I would have been hungry from the moment I opened my eyes to the moment I closed my eyes, but I wasn't. (Woman, aged 42 years, 1 year since diagnosis).

Only a few interviewees reported having deviated from the diet. This makes it harder to draw conclusions about what the situations are in which there is a higher risk of lapsing, and what strategies are effective in facilitating adherence in such situations. For example, the participant who did not manage to lose the required amount of weight in the beginning of the study struggled after the VLED because he changed environments early on and found it difficult to manage triggers such as smell of food.

Because the smells of people eating all around you, I was in town at one point, bakeries everywhere and, it was ridiculous, I couldn t concentrate…I would have been fine if I had been at home, I would have lost weight this week and I would have still been on it, but I couldn't stick to it. (Man, 52 aged years, 1 year since diagnosis).

The limited data also suggest that physical and social environments, together with emotional states and availability of self‐regulatory skills were the most important factors affecting adherence. Feelings of sadness, loneliness or stressful experiences affected the participants’ willpower and increased the attractiveness of a temptation, as illustrated by the quote below.

I was so frazzled I went up to the desk and there was some chocolates right in front of us [me] and I was so tempted to have one but I walked away from them, although I have to say I walked up to the tin and lifted the lid three times but each time I just walked away from it because I thought no, because that would have felt like I had given in and I didn't want to do that. (Woman, aged 35 years, 1.5 years since diagnosis).

Awareness, weighing of pros and cons of giving in, conscious deliberation and often self‐talk and reminding oneself of one's goal were crucial to behaviour‐regulation in tempting situations. When it came to actual dietary deviations, these were the consequence of both an impulse triggered by the presence or smell of food and a reflection, i.e. a deliberation and a subsequent conscious decision to go off the diet.

Our observations based on the limited data suggest that it was situations in which the participant was alone when they were tempted or gave in, and this may be attributable to the social pressure the participants had created by broadcasting taking part in the study at the start of the VLED, and not wanting to fail in front of other people.

### Facilitators of adherence

Rapid results, social support and involvement in a research study were the main facilitators of adherence. Seeing physiological changes quickly facilitated reinforcement of behaviours supporting adherence.

I think it's been the initial the weight loss, how quick it's gone off, but obviously that's because of the very‐low‐calorie diet, but I think that's the bit that's motivating us [me] to continue, that I've done two and a half stone now and it's not impossible to do another two and a half stone but it will possibly take longer than this has. (Woman, aged 47 years, 2.5 years since diagnosis).

Changes in weight were soon reflected in changes in clothing size, providing additional motivation to keep up. The change in clothing size boosted the participants’ confidence, feelings of well‐being and sense of attractiveness, and it also served as a way of measuring their weight loss success.

Receiving compliments on appearance was one of many ways participants felt supported during the VLED. Some relatives of the participants had altered their own eating behaviours to help facilitate adherence of the participant. This included eating at different times, refraining from offering food, warning the participants before cooking, so that they could engage in another activity or avoid the environment, reminding the participants of what they were or were not allowed to eat, and even embarking on their own weight loss alongside the participant's. Having a friend or a relative who had gone through the same or a similar weight loss programme and who had given them advice and words of encouragement also helped the participants’ preparedness and motivation before and during the VLED. In addition, creating a support system with friends or relatives who were losing weight at the same time was helpful for the purposes of experience‐sharing, encouragement and sometimes competition.

Because of the limited number of people planned to be recruited into the Counterbalance study, the participants considered taking part a unique opportunity. Not wanting to disappoint the study staff or distort results for the study were strong drivers of adherence. The participants were aware of the effort and time the study staff had put into the running of the study, the provision of ongoing individualized support, and the potential future implications of the study on treatment of diabetes. The awareness contributed to the participants’ sense of shared effort for a common purpose and provided further motivation to adhere to the VLED.

I do recognize the fact that this is a medical study so I'm one of a handful of people that are lucky to actually be on it so to not do as what I'm told would just be silly, stupid. (Man, aged 49 years, 9.5 years since diagnosis).

### Behaviour regulation

In order to maximize adherence and overcome the identified barriers to it, the participants developed and employed behaviour‐regulation strategies before and during the VLED (a detailed list of strategies the participants and their relatives used to facilitate adherence is given in Tables 4 and 5). The main strategies were removing food from the environment, planning, avoidance of tempting situations or places, and self‐distraction, and they were often used in combination. Removing food from the participants’ usual environments was usually the first and immediate strategy employed. This included throwing or giving away, or eating up all leftover food before the study commenced, and asking colleagues not to offer them any treats during the 8 weeks. Preparation included cooking soups and vegetables in batches and freezing them, so that they would be ready to be eaten after coming home from work, while planning portions for the day, and carrying water and vegetables in pockets or bags often helped with cravings.

**Table 4:** List of strategies that the participants found helpful for their adherence to the very‐low‐energy diet

Group	Strategy
Food removal	Throwing away/giving away/eating up leftovers before starting the VLED. Not buying undesired food. Keeping undesired food out of sight. Freezing undesired food so that it's not immediately available during a craving.
Avoidance of…	…places where there is limited choice or lack of healthy food options. … television watching, in order to avoid looking at food adverts or habitual snacking. …social events with abundance of food. …shopping in shopping centres; shopping can be done online instead. …eating with other people.
Planning	Planning the logistics of being on a diet; e.g. food shopping/cooking/eating times, attendance of social events, and coming to terms with the plan. Thinking about and preparing food for the next day. Cooking in batches and freezing food for quick healthy meals. Having healthy nibbles at hand (e.g. carrot sticks, pieces of apple etc.). Carrying a bottle of water.
Hunger management	Drinking water throughout the day. Drinking water when starting to feel hungry. Spreading meals throughout the day. Adding spices and herbs to the VLED shakes to increase variability and palatability. Drinking the VLED shakes hot or very cold to increase palatability. Getting active/distracting oneself from thinking about food (e.g. gardening, hobbies). Adding more water to the VLED shakes to increase volume. Chewing a gum or a sugar‐free mint. Going to bed earlier. Allowing oneself a taste of food to satisfy curiosity and prevent cravings. Self‐talk and negotiation when tempted, weighing the pros and cons of eating undesired food. Reminding oneself of one's goals. Reminding oneself of one's success. Becoming aware of situations in which one feels tempted. Being kind to oneself after a lapse and carrying on with the plan.
Social	Telling other people about one's weight loss attempt to prevent temptations from others, to get support and understanding, and to increase the commitment to one's weight loss plan. Getting a weight loss ‘buddy’ to share experiences and tips with, to be accountable to, and to facilitate adherence (this would ideally be a partner). Getting monitored by a third party, e.g. asking one's general practice for regular weigh‐ins.

VLED, very‐low‐energy diet.

**Table 5:** List of behaviours of other people that the participants found helpful for their adherence to the very‐low‐energy diet

Giving compliments on effort, appearance and energy. Eating at different times. Refraining from offering food to the participant. Giving the participant notice before cooking. Reminding the participant of what they are or are not allowed to eat and drink. Embarking on their own weight loss alongside the participant. When asked, giving advice from relevant experience. Encouraging the participant to keep going. Cooking meals for oneself or getting ready meals if the participant is the main cook. When cooking, leaving pieces of vegetables on the side for the participant to nibble on to curb temptations and cravings. Not buying unhealthy food. Joining the participant in non‐food related activities. Employers enabling time off work for regular appointments. Healthcare professionals: providing regular monitoring of weight and blood glucose levels. Healthcare professionals: providing physical feedback on the participant's health outcomes (e.g. graphs, scans etc.). Healthcare professionals: providing individualised behavioural support. Healthcare professionals: explaining in detail any queries the participant may have in relation to the diet and their health. Healthcare professionals: being available to respond to queries by telephone or e‐mail in‐between appointments if needed.

I made sure that I had all my three shakes and I think I put some of the peppers or carrots in a little bag so I was able to nibble those. (Woman, aged 70 years,15 years since diagnosis).

Similar to food removal and some of the preparatory strategies, avoidance strategies were part of restructuring of the participants’ immediate environments. It included avoidance of activities related to snacking (e.g. watching television) and places where there was lack of choice of healthier food options (e.g. pubs), or avoidance of eating with others (e.g. work lunches). Use of this strategy decreased with progression of the diet because of the early motivational boost related to changes in weight and blood glucose levels, and increase in the participants’ behaviour regulation self‐efficacy. Lastly, distraction was often associated with avoidance, and used when avoidance was not always possible, or as a complementary strategy to it. Distraction in the form of engaging in alternative activities and by keeping busy in general was used to divert one's attention from thinking about food when hunger levels increased, whether or not food was present. Going for a walk, gardening, or reading were the most prevalent alternative activities. The participants also spent more time on hobbies and reduced the time spent watching television because of being in the habit of snacking while watching, or because they did not want to be tempted by adverts promoting food.

Because if you sit still for 5 minutes or you're watching one of your chick flicks or whatever the thing is oh popcorn would be nice but you just I just don't sit and watch any movies. I went and mowed the lawn. (Woman, aged 42 years, 1 year since diagnosis).

### Changes in physical and psychological well‐being

The amount of weight loss, improvements in blood glucose and physical fitness, compliments from other people, and the participants’ overall sense of confidence, control and achievement all contributed to their perception of improved psychological well‐being and happiness. Most participants found that their mood remained stable, despite their anticipation of irritability or grumpiness. They gradually felt better about themselves, felt happier, less ill, more optimistic about their future, and they were pleased with the change in their body shape.

I now realise that I feel better inside, not just looking better on the outside… I just feel I don't feel ill and that's how I felt before. (Woman, aged 42 years, 1 year since diagnosis).

The participants have also become more sensitive to their feeling of hunger, learned how their body responds to calorie restriction and food, and about their behaviours in situations in which they felt tempted, which helped them better understand their relationship with food.

It's made me more conscious of where extra calories slip in, especially on the ward when they have huge tubs of chocolates on the desk every day. Or huge tins of biscuits. (Woman, aged 35 years, 1.5 years since diagnosis).

Physical well‐being also changed during the VLED. Although the participants’ energy levels dropped initially as a result of the calorie restriction, they improved gradually over the 8 weeks. The increased levels of fitness were reported as being able to do the same activities for longer or at a higher intensity, and being able to function better in their daily life by engaging in activities such as walking, climbing stairs, doing work around the garden, or playing with grandchildren.

It was fairly hard to start with but it got easier as the weeks went on and then when I started getting a bit fitter and I could walk further and stand up and sit down and dig the garden it's great now. I feel great. (Man, aged 44 years, 2.5 years since diagnosis).

### Acceptability of the VLED

The simplicity of the meal replacement preparation and the regimen of the diet was appreciated, as participants did not have to spend much time shopping for food, preparing food, or making decisions about either, and because they knew exactly what they were to eat every day.

Well a simpleton could follow it because there's not a lot of food and lot to do with it. Add cold water to this and that's it. (Man, aged 67 years, 3.5 years since diagnosis).

The different flavours and consistency of the sachets were generally well received. Although the first 2–3 days on the diet were the most challenging, the initial 2–3 weeks were often perceived as easier than expected with regard to levels of hunger and getting used to the diet. This tended to change mid‐way through the VLED, when participants seemed to get used to the regimen and started becoming bored. At this stage, the diet was often perceived as tedious or monotonous, which became more apparent within the last 2–3 weeks as a result of lack of variability of the food allowed and lack of solid food.

Towards the last week and a half I got bored. Boredom set in but that was all. I mean boredom in the sense of…just lack of variety, It's just the fact that it's the same day after day…It's all of that that was more difficult, but I wouldn't like to overstate it because it's only 8 weeks. It's not like it's a big chunk of your life is it really?’ (Man, aged 59 years, 10 years since diagnosis).

This was, in most cases, overcome by more experimentation with flavours and by putting the length of the VLED phase into a time perspective, acknowledging that it was only a short period in their life that would potentially result in long‐lasting health benefits.

### Most useful study features and suggested improvements

Receiving bio‐feedback and waiting to start on the VLED were the most useful (although not always intended) study features. Bio‐feedback was provided repeatedly during the 8 weeks and included: feedback on anthropometric measures (e.g. weight, waist circumference) at baseline and weeks 1, 4 and 8, and an MRI scan of the pancreas and liver at baseline and at week 8. In addition, the participants were given regular individualized support with achievements of their goals throughout the diet during planned visits and as‐needed. The bio‐feedback as well as the individualized support provided additional motivation to adherence.

Most participants would have welcomed an opportunity to meet other participants in order to exchange their experience and tips, compare their progress and socialize with people who were going through the same programme.

I think comparison helps…It's about how am I doing in relation to other people. Am I doing better than other people because that's always nice to know if you are. (Man, aged 59 years, 10 years since diagnosis).

Some participants suggested inclusion of food variations or alternatives (vegetables, fruits, spices) in the information sheet as a guidance. This would prevent them from choosing unsuitable ingredients and prevent boredom with the VLED regimen.

## Discussion

**Figure 3:**
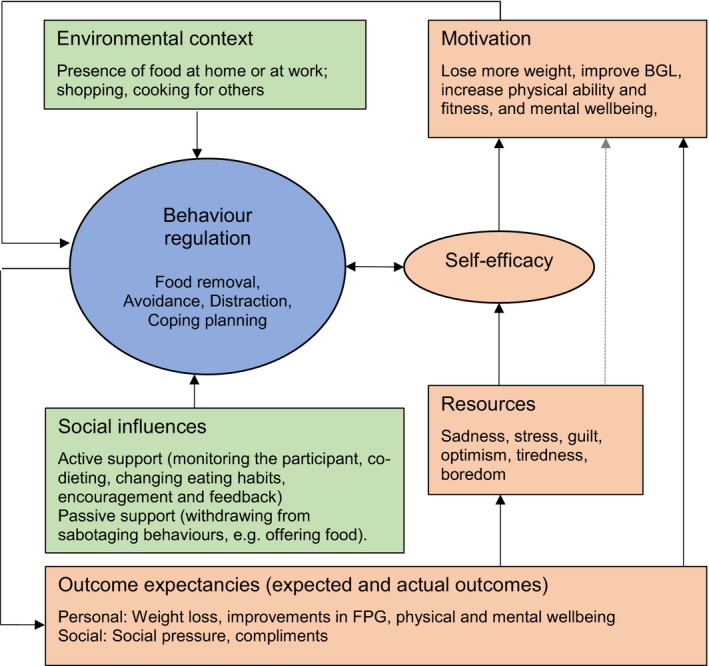
Model of psychological, behavioural and environmental determinants of adherence with the very‐low‐energy diet, based on baseline and follow‐up interviews. Highlighted in pink are psychological determinants; highlighted in green are environmental determinants; highlighted in blue are behavioural determinants. Full line represents relationships substantially supported by data; dotted lines represent relationships that need further exploration. Direction of the relationship is hypothesized.

Among the participants of the present clinical study, the VLED for diabetes remission was perceived as highly acceptable and easier to adhere to than the participants had anticipated. The identified barriers to adherence were, in reality, minimally disruptive to adherence, and these were feelings of hunger, emotional distress and environmental triggers, while rapid outcomes, social support and involvement in a research study facilitated adherence. To overcome the perceived barriers, people used four main behaviour‐regulation strategies: removing food from the environment; avoidance; self‐distraction; and planning. The taste and structure of the VLED intervention was well accepted. The participants suggested inclusion of more flavours in the diet and meeting other participants for support. Figure 3 shows a model of psychological, behavioural and environmental determinants of adherence to the VLED, based on the baseline and follow‐up interviews, and informs discussion.

While diabetes reversal and large weight loss were the main motives for the participants to take part in the Counterbalance study, an array of expectations and values related to health, family, quality of life or appearance were important too. The significance of improved body image and appearance through weight loss was identified in previous qualitative studies 20, 21 and, together with health improvement, these are the major motives for weight loss 21, 22, 23, 24, potentially because of the perception that ‘beautiful’ often means ‘healthy’ 25. The amount of weight lost in the Counterbalance study led to higher perceived self‐confidence and physical and psychological well‐being, which facilitated the continuous motivation to adhere to the VLED. This is consistent with findings from another study in which the main motivation for participants to enrol in the weight‐loss programmes was lack of self‐esteem and confidence, and the expectation of increased intrinsic sense of worth through weight loss 20.

The National Weight Control Registry in the USA reported that 83% of its members experience a ‘trigger’ for their weight loss, such as medical reasons (23%), reaching an all‐time highest weight (21.3%), and their reflection in the mirror (12.7%) 26. In the present study, the motivation to lose weight was often triggered by life events or ‘life crises’ 27, such as the shock of diagnosis, having seen the consequences of Type 2 diabetes in other people and wanting to avoid them, or trying to conceive a baby. Many people attempting to lose weight may therefore have health‐related motives, which are also associated with better weight loss outcomes, than appearance‐related motives 28. This may have implications for future studies because appearance‐related motives for weight loss tend to be positively associated, while health‐related motives are negatively associated with body image concerns 23.

The theme of social influence was central to the narratives of the participants and it may explain why social support often helped override environmental or emotional triggers, and why one of the suggestions for improvement was getting a chance to speak to other participants.

The limited data on lapses in the present study indicate that low level of positive resources was associated with some of the situations in which the participants felt most tempted or when they deviated from the VLED. This is consistent with the ego‐depletion hypothesis 29, according to which the ability to self‐regulate decreases with prolonged effort. When self‐regulation resources are low, people tend to switch to automatic attitudes and behaviour, and risk a behavioural lapse 30. Our data suggest that high resources (e.g. increased energy levels, optimism and confidence) had a positive impact on the participants’ continuous motivation and their ability to adhere to the VLED. Only a handful of lapses were reported during the follow‐up interviews and they were considered an exemption from a rule. They were mostly planned ahead and not regretted, which may have helped prevent feelings of guilt or shame, and may also be the reason why they did not seem to negatively affect self‐efficacy. Moreover, previous research found that subjective ratings of appetite decrease significantly during ketosis induced by a VLED in people without diabetes as a result of diet‐induced changes in concentrations of appetite‐stimulating hormones and nutrients 31, which may partially explain the overall low levels of reported hunger and the number of lapses in the present study. The low number of reported lapses, may mean that there were no additional lapses, or that they stayed unreported because of feelings of shame, disappointment, fear of being found out about, or self‐presentation bias 32, preventing us from drawing conclusions about the effect of low resources on adherence to the VLED.

Past responses to social and environmental contexts during the VLED seemed to affect the participants’ belief in their ability to adhere to the diet (self‐efficacy) most. Successful behavioural responses (e.g. being able to take a shake to a restaurant or attend an event where food was present and not deviating from the diet) reinforced adherence self‐efficacy, which fed future behaviour regulation, increasing the chance of perseverance in trying to achieve the goals. This finding corresponds to the concept of mastery experience within Social cognitive theory 33, which proposes that the most influential source of self‐efficacy is one's previous performance. We identified a number of behaviour regulation practices the participants used to overcome challenging situations and that other people used to facilitate the participants’ efforts (Tables 4 and 5). Use of similar dietary, physical‐activity, cognitive and tracking practices during weight loss have been previously identified in a cross‐sectional survey that examined the association between the practices people used in the past week and their success in weight loss (≥ 10%) in the past year 34. Out of 36 identified practices, only 18 were associated with successful weight loss. While there is some overlap with the practices we have identified and those found by Sciamanna *et al*. 34, some practices were not identified by their study. This may be attributable to the VLED being qualitatively and experientially different from other weight loss approaches, which means that people going through it may use slightly different practices and have different support needs during the weight‐loss process.

Although the number of participants recruited into the Counterbalance study via each of the recruitment channels was not recorded, the information was extracted from the interviews and was available for 17 out of the 18 participants. Out of these, six participants were recruited through word of mouth. Nine of the 18 participants who finished the study were ‘buddies’ to each other or had a ‘diet buddy’ not involved in the Counterbalance study. Having a diet buddy during the intervention may have provided crucial social support and may also explain the excellent completion, reported adherence, weight loss and diabetes remission rate. Higher levels of social support are associated with improved diabetes and behavioural outcomes, although acceptable levels of social support may vary between men and women. While support from a spouse may have positive effects on weight reduction in obese women with Type 2 diabetes, it does not have the same effect on men 35. Similarly, high satisfaction with social support has a positive impact on diabetes control for women, but not men 36, which may need to be taken into consideration when tailoring support to individuals with Type 2 diabetes. Recruiting participants with friends increases the availability of social support, and results in higher rates of weight loss treatment completion as well as better weight loss maintenance 37. Finally, the regular monitoring and individual feedback on weight and diabetes outcomes from the study staff were substantial facilitators of adherence to the VLED in the present study and a well‐documented phenomenon in a number of systematic reviews 38, 39, 40, 41. Individuals who self‐weigh regularly lose more weight and also consume fewer calories than people who do not monitor their weigh regularly 42. Furthermore, regular appointments, recognizing responsibility, positive attitude and support from others and dieticians are also key in promoting weight loss 39, which is much in line with our findings on the importance of social support and the importance of being involved in a medical study with regular monitoring.

The present study provides a qualitative account of peoples’ experiences with a VLED and its acceptability, complementing the existing quantitative evidence about efficacy of the VLEDs. We have identified the main barriers and facilitators of adherence to a VLED, as well as behavioural strategies that people use to overcome them. Findings from this study can be used to inform interventions to support people during a dietary diabetes remission programme in the future, as well as estimate the human, time and physical resources needed for successful implementation of such an intervention. This study was developed and conducted in collaboration with an experienced multidisciplinary team of researchers and clinicians, providing more confidence in interpretation of the results.

The following limitations need to be taken into consideration, however, when interpreting the results of this study in terms of generalisability. Firstly, the Counterbalance study was conducted on university premises, with a well‐equipped magnetic resonance centre and with devoted resources. The qualitative data showed that being involved in a research study itself provided substantial motivation to adhere to the VLED, which has been found in other studies too 20. The provision of this level and intensity of support, care and feedback, however, is unlikely in a primary care setting, because of practical constraints. Secondly, participants who had a ‘diet buddy’ might have had *a priori* social support in place, potentially increasing their initial level of motivation beyond that which would be found in the general population. Thirdly, linked to the involvement in a research study is the extent to which the participants were willing to disclose their honest opinion of the VLED itself, or share experience with their dietary temptations and deviations. Because lapses were rarely reported, it is possible that the participants may not have disclosed all relevant experience with regard to the VLED because of fear of being found out or self‐presentation bias 32, even though all interviews were confidential and anonymized. Lastly, recruitment for the qualitative study started halfway through the timeframe of the Counterbalance study, and therefore not all participants could be interviewed. Although interviews with the rest of the participants might have brought up additional themes, data saturation was reached quite early into follow‐up interviews, hence it is unlikely that important themes have been missed out. Future studies could replicate the present study with a sample of patients recruited through general practice lists to ensure higher heterogeneity of participant characteristics, which could potentially bring up themes that may not have been brought up by our sample.

Although the present study showed high levels of adherence and acceptability of the VLED, its limitations suggest that less favourable outcomes may be achieved in a primary care setting, with fewer resources and less‐motivated individuals. Future studies conducted in a routine clinical care setting with usual level of resources could assess the effectiveness of the use of VLEDs for weight loss and Type 2 diabetes remission and provide more evidence for health service providers, with potential extensive public health implications.

In conclusion, dietary Type 2 diabetes remission through a VLED is acceptable and feasible in motivated participants and the process is perceived as highly gratifying. Research outside of controlled trial settings is needed to gauge the generalisability of these findings.

## Funding sources

L. R. was funded by a PhD fellowship from the Institute of Health and Society, Newcastle University at the time of the study. A. A. is funded by a UK National Institute of Health Research Professorship. F. F. S. is funded by Fuse, the UK Clinical Research Collaboration Centre of Excellence for Translational Research in Public Health. Funding for Fuse from the British Heart Foundation, Cancer Research UK, Economic and Social Research Council, Medical Research Council, and the National Institute for Health Research, under the auspices of the UK Clinical Research Collaboration, is gratefully acknowledged. The funders had no influence on the research reported in this paper.

## Competing interests

The Counterbalance study (‘Characterization of the principle determinants of long‐term reversal of Type 2 diabetes’, REC 12/NE/0208) was funded by the Novo Nordisk UK Research Foundation and Newcastle National Institute of Health Research Biomedical Research Centre funding grant. L. R., A.A., F.F.S. and R.T. are currently engaged on a research project using VLEDs for diabetes remission (DiRECT) funded by Diabetes UK. The authors have no other conflicts of interest to declare.

### Prior presentation

Part of this study was presented at the annual conference of the Australian Psychological Society in 2015.
